# Wage gaps between US nurses with and without disabilities

**DOI:** 10.1093/haschl/qxag161

**Published:** 2026-06-27

**Authors:** Laurin Bixby, K Jane Muir, Michelle Kephart, Mihir Kakara

**Affiliations:** Lurie Institute for Disability Policy, The Heller School for Social Policy and Management, Brandeis University, Waltham, MA 02453, USA; Center for Health Outcomes and Policy Research, School of Nursing, University of Pennsylvania, Philadelphia, PA 19104, USA; The Leonard Davis Institute of Health Economics, University of Pennsylvania, Philadelphia, PA 19104, USA; Department of Emergency Medicine, Perelman School of Medicine, University of Pennsylvania, Philadelphia, PA 19104, USA; Department of Mentoring, Leavitt School of Health, Western Governors University, Salt Lake City, UT 84107, USA; Department of Leadership, Research, and Foundations, University of Colorado, Colorado Springs, CO 80918, USA; Department of Neurology, NYU Grossman School of Medicine, NewYork, NY 10016, USA

**Keywords:** disability, nursing, workforce

## Introduction

Wage disparities between workers with and without disabilities are well-documented, with evidence implicating employer discrimination and job requirements as underlying factors.^1^ However, there is limited evidence regarding disability-based wage gaps among nurses—the largest clinician group in the U.S.^2^

Disability and health are cited as key reasons for nurse job dissatisfaction,^2,3^ including inadequate work accommodations and hostile work environments.^4^ Employers face challenges recruiting and retaining nurses, and disabled nurses are more likely to leave the workforce.^5^ Challenges recruiting and retaining nurses highlight the urgency of improving work environments and compensation practices to attract nurses into employment, including those with disabilities.^3^

Most nursing workforce policies in the U.S. focus on promoting supportive work environments, such as minimum nurse staffing requirements,^2^ with less attention to disability-inclusive workplace policies. Further, while prior literature demonstrates racial/ethnic wage disparities among nurses^6^, disability-related wage disparities remain understudied. Characterizing these disparities is important for contextualizing policies and workplace practices that support nurse well-being and workforce retention.

## Methods

This study analyzed cross-sectional data from the 2008-2023 American Community Survey (ACS) among working-age (20-65 years old) employed nurses. Nurses self-identified as Registered Nurses (RNs) or Advanced Practice Registered Nurses (APRNs): Nurse practitioners, Nurse anesthetists, and Nurse midwives. An individual was considered disabled if they answered yes to any of the 6 ACS disability questions (Appendix 1). Linear regressions estimated outcomes (logged annual and hourly wages and total hours worked annually) as a function of disability, adjusting for age, sex, race/ethnicity, year, and state-by-metropolitan area fixed effects. We conducted subgroup analyses by occupation (RNs vs APRNs), age-group, and disability type. All models included robust standard errors and incorporated ACS survey weights and complex survey design.

## Results

The analytic sample included 473 850 employed nurses, with 17 425 (weighted, 3.5%) reporting a disability. See Table S1 in Appendix 2 for sample characteristics. The most common disability types were mobility (23.9%) and hearing (22.1%) disabilities. Employed, working-age nurses with disabilities were older, on average, than those without disabilities (mean age: 48.6 vs 42.6, respectively). The top industries where nurses with and without disabilities worked were overall similar (ie, hospitals, outpatient care centers, and nursing facilities), though a smaller proportion of disabled nurses (55.1%) worked in hospitals than non-disabled nurses (63.7%).

In adjusted analyses, disabled nurses earned 12.2% lower annual wages (95% CI, −13.6 to −10.8; *P* < 0.001) and 7.4% lower hourly wages (95% CI, −8.3 to −6.5; *P* < 0.001), and worked 27 fewer hours annually than non-disabled nurses. Wage differences between nurses with and without disabilities remained significant after adjusting for total hours worked (Table 1). Models that additionally adjusted for industry (job location) showed similar estimates for primary outcomes (see Table S2 in Appendix 2).

**Table 1.** Differences in earnings and total hours worked for employed nurses with a disability compared to nurses without a disability^a^.

	Adjusted Models			Models additionally adjusting for total hours worked		
	Estimate	95% CI	*P* value	Estimate	95% CI	*P* value
Difference in Annual Wages^b^, percentage points^c^	−12.2	−13.6, −10.8	<0.001	−10.5	−11.7, −0.9.4	<0.001
Difference in Total hours worked (annual)^d^	−26.8	−38.8, −14.8	<0.001	NA	NA	NA
Difference in Hourly Wages^e^, percentage points^c^	−7.4	−8.3, −6.5	<0.001	−7.7	−8.6, −6.8	<0.001
Subgroup analyses for occupation subgroups^f^:						
Registered Nurses: Difference in Annual Wages^b^, percentage points^c^	−11.6	−13.1, −10.1	<0.001	−10.1	−11.3, −8.8	<0.001
APRNs: Nurse Practitioners, Nurse Anesthetists and Nurse Midwives: Difference in Annual Wages^b^, percentage points^c^	−6.0	−11.5, −0.5	0.033	−5.9	−10.5, −1.3	0.013
Subgroup analysis by age group: Difference in Annual Wages^b^, percentage points^c^						
Age 20-34	−10.6	−14.2, −7.1	<0.001	−9.9	−12.8, −7.0	<0.001
Age 35-49	−12.8	−15.5, −10.2	<0.001	−11.6	−13.8, −9.4	<0.001
Age 50-65	−12.1	−13.9, −10.2	<0.001	−10.1	−11.6, −8.6	<0.001
Analyses by disability type: Difference in Annual Wages^b^ compared to those without disability, percentage points^c,g^						
Cognitive	−18.8	−22.8, −14.8	<0.001	−14.1	−17.3, −10.8	<0.001
Mobility	−12.5	−15.4, −9.7	<0.001	−11.2	−13.5, −8.9	<0.001
Independent living	−17.9	−25.5, −10.3	<0.001	−10.2	−16.9, −3.4	0.003
Self-care	−7.9	−17.8, 2.0	0.12	−6.5	−15.6, 2.6	0.16
Vision	−4.9	−7.7, −2.2	<0.001	−9.2	−11.7, −6.7	<0.001
Hearing	−0.9	−3.3, 1.4	.43	−1.6	−3.6, 0.4	.12
Multiple disabilities	−26.3	−30.3, −22.2	<0.001	−19.0	−22.1, −15.8	<0.001

Abbreviations: CI, confidence interval; NA, Not applicable.

^a^All outcomes were analyzed for employed nurses aged 20-65, using linear regression models with robust standard errors. Models were adjusted for fixed effects for age (individual years), sex (male, female), race (White, Black/African American, American Indian or Alaskan Native, Asian or Pacific Islander, Multiracial, Other), ethnicity (Hispanic, Not Hispanic), state and metropolitan area, and survey year.

^b^Annual wage denotes income earned from wages as an employee for the previous year. It is the total pre-tax wage and salary income. Sources of income include wages, salaries, commissions, cash bonuses, tips, and other money income received from an employer.

^c^Because logged wages were used as the dependent variable, estimated earnings disparities can be expressed as percent differences (coefficient multiplied by 100).

^d^Total hours in a year worked are calculated by multiplying hours worked per week and weeks worked in the year.

^e^Hourly wages calculated by dividing annual wages by total hours worked in the year. Total hours worked is calculated by multiplying hours worked per week and weeks worked in the year.

^f^ACS started dividing nursing professionals into “Registered Nurses”, “Nurse anesthetists”, and “Nurse practitioners and Nurse midwives” starting from 2010. These analyses reflect years 2010 to 2023.

^g^Analyses by disability type compare respondents with only the listed type of disability vs those without any disability. Respondents with more than one disability type are included under the “Multiple disabilities” type.

Significant annual wage differences by disability existed for RNs and APRNs, with larger gaps among RNs (11.6%; 95% CI: −13.1 to −10.1; *P* < 0.001) than among APRNs (6.0%; −11.5 to −0.5; *P* < 0.001). Age-stratified analyses showed similar significant wage differences across age groups. The magnitude of wage gaps varies across disability types, with the largest differences among nurses with cognitive, mobility, and multiple disabilities.

## Discussion

Our findings indicate significant associations between disability and wages among employed nurses, with disabled nurses earning significantly lower wages than non-disabled nurses. A natural question is whether this reflects individual choice (eg, trading pay for job flexibility) vs broader policy and organizational factors that create wage gaps. While both may play a role, our findings and prior research suggest an important role of the latter. Wage gaps persisted after adjusting for hours worked and across age groups, occupations, and disability types, suggesting that workplace factors, rather than occupational distribution (ie, disabled nurses self-selecting into certain occupational phenotypes), potentially underlie these findings.

Moreover, individual “choices” are frequently constrained by organizational factors. Lack of workplace accommodations, inadequate flexibility, and insufficient managerial support can restrict career advancement or push nurses into lower-paying roles or to leave the profession.^4^ Prior studies show that disabled nurses and students frequently encounter discrimination and unsupportive environments and cite disability or health as key reasons for job dissatisfaction.^4,7^ More broadly, discrimination is a well-documented contributor to disability-related wage gaps.^1^ Future research should explore how systems-level factors, including access to accommodations, influence wage disparities, which can exacerbate job dissatisfaction, burnout, nursing turnover, and understaffing, with downstream effects on patient care.^3,4^

Strategies to support disabled nurses should create safe, supportive work environments and organizational practices, including job flexibility, manageable workloads, and opportunities for mentorship and advancement.^4^ This includes shifting standards toward task completion with appropriate accommodations rather than functional ability alone, establishing clear processes for accommodations, and ensuring safe disability disclosure without fear of stigma or discrimination.^7^ Addressing ableist attitudes through education and improving awareness of legal rights and disability accommodations are also essential.^4,8^

Potential organizational approaches to create more inclusive work environments include the American Nurses Credentialing Center Magnet**®** Hospital program, a designation representing an institutional commitment to high-quality nursing work environments with supportive nurse leaders and nurse autonomy. Magnet® Hospitals provide more inclusive work environment cultures committed to equitable employer policies.^9,10^

Additionally, emerging technology-based care delivery models, such as virtual nursing care, should be explored. Virtual nurses augment bedside nurses’ care by conducting hospital admissions, medication reconciliation, blood administration checks, and patient education. Such models, though still early in implementation and pending robust outcomes data,11 may provide more accessible roles for disabled nurses without compromising wages.

Study limitations include that ACS undercounts mental health, communication, learning, and episodic disabilities, potentially biasing results towards the null. Further, nurses with severe disabilities and/or facing workplace-related barriers who have left the workforce are not captured in this sample, potentially underestimating wage disparities. Lack of data on disability onset, severity, cause, and career stage limits understanding of drivers of wage gaps and may contribute to residual confounding.

Overall, these findings point to wide disability-based wage gaps within the largest U.S. healthcare workforce. As more nurses enter the workforce with disabilities or acquire them with age or the physical demands of the job,^3^ policy and organizational changes to foster disability-inclusive workplaces are essential to improve retention, equity, and patient care.^4^

## Supplementary Material

qxag161_Supplementary_Data

## References

[1] Kruse D, Schur L, Rogers S, Ameri M. Why do workers with disabilities earn less? Occupational job requirements and disability discrimination. Br J Ind Relat. 2017;56(5):798–834. 10.1111/bjir.12257

[2] Smiley RA, Kaminski-Ozturk N, Reid M, et al The 2024 national nursing workforce survey. J Nurs Regul. 2025;16(1):S1–S88. 10.1016/s2155-8256(25)00047-x

[3] Muir KJ, Porat-Dahlerbruch J, Nikpour J, Leep-Lazar K, Lasater KB. Top factors in nurses ending health care employment between 2018 and 2021. JAMA Netw Open. 2024;7(4):e244121. 10.1001/jamanetworkopen.2024.4121 38592723 PMC11004833

[4] Baker C, Malik G, Davis J, McKenna L. Experiences of nurses and midwives with disabilities: a scoping review. J Adv Nurs. 2023;79(11):4149–4163. 10.1111/jan.15802 37553870

[5] Ly DP . Age, disability, and household composition of nurses and physicians who are not in the labor force. PLoS One. 2021;16(2):e0247967. 10.1371/journal.pone.0247967 33635918 PMC7909633

