# Supplementary material for: Wage gaps between US nurses with and without disabilities
Source: Health Aff Sch. 2026 Jun 27;4(7):qxag161. doi: 10.1093/haschl/qxag161 (PMC13370830; doi:10.1093/haschl/qxag161)
Supplement: qxag161_Supplementary_Data [file qxag161_supplementary_data.zip › FINAL_HA_Scholar_Supplementary_Material_Kakara_R&R_CLEAN.docx]

**Supplementary Material**

**Appendix 1:**

**Methods**

**Data Source**: The American Community Survey (ACS) is a nationally representative, annual cross-sectional survey conducted by the U.S. Census Bureau. The publicly available ACS data were obtained through the Integrated Public Use Microdata Series (IPUMS) database.^1^

**Identification of sample**:

Nursing professionals were identified based on reported occupation, which was represented by a single code until 2009. Beginning in 2010, separate occupation codes were introduced for “Registered Nurses,” “Nurse Anesthetists,” and “Nurse Practitioners and Nurse Midwives.” The latter two categories were combined and classified as Advanced Practice Registered Nurses (APRNs).

Nurses were identified to have a disability if they answered Yes to any of the standard 6 disability questions in the ACS:

1) Because of a physical, mental, or emotional condition, does this person have serious difficulty concentrating, remembering, or making decisions?

2) Does this person have serious difficulty walking or climbing stairs?

3) Because of a physical, mental, or emotional condition, does this person have difficulty doing errands alone such as visiting a doctor's office or shopping?

4) Does this person have difficulty dressing or bathing?

5) Is this person blind or does he/she have serious difficulty seeing even when wearing glasses?

6) Is this person deaf or does he/she have serious difficulty hearing?

For analyses by disability type, we compared nurses with only the specific type of disability versus those without any disability. We report a separate category of ‘Multiple disabilities’, where respondents have more than one type of disability. Of note, ACS is known to undercount disability, especially individuals with mental health, intellectual and communication disabilities.^2^

Only those reporting at least 2 years of college-level education were included, along with a lower age cut-off of 20 years to include those who had complete 2-year nursing programs. An upper age limit of 65 years was selected to align with the traditional retirement age and to reduce potential bias from age-related factors. Nurses who remain employed after age 65 may represent a selective group with different work patterns, health status, disability experiences, retirement decisions, and compensation structures than younger nurses, and would introduce heterogeneity related to retirement-related factors

**Variables:**

**Race:** All self-reported race categories from the ACS were included: White, Black/African American, American Indian or Alaska Native, Asian or Pacific Islander, Other race not elsewhere classified (nec), two major races, and three or more major races (the latter two were combined into a “Multiracial” category).

**Ethnicity:** Ethnicity was categorized as Hispanic or Not Hispanic. Among Hispanic respondents, Cuban, Mexican, and Puerto Rican ethnicities were reported separately. The “Other Hispanic” category included Costa Rican, Guatemalan, Honduran, Nicaraguan, Panamanian, Salvadoran, Argentinian, Bolivian, Chilean, Colombian, Ecuadorian, Paraguayan, Peruvian, Uruguayan, Venezuelan, Spaniard, Dominican, and other Hispanic origins not listed above.

Both race and ethnicity were self-reported by ACS participants. For state fixed effects, individual states were identified using Inter-University Consortium for Political and Social Research (ICPSR) codes in the ACS. For metropolitan area fixed effects, areas were classified as metropolitan, mixed metropolitan, or non-metropolitan.

For industry classifications, the Census industrial classification system was used. Relevant to the data shown, ‘hospitals’ and ‘general medical and surgical hospitals’ were combined into one category (Hospitals), and ‘Outpatient care centers’ and ‘Offices of Physicians’ were combined into one category.

**Labor Force Participation:** As defined by the U.S. Census Bureau in the ACS, the labor force participation includes people both currently working or seeking work. Employment status indicates whether a person is currently working versus not (does not include those seeking work). This study includes nurses who are only currently employed. Among working-age nurses (ages 20-65 years old), labor force participation was lower for disabled nurses (70.8%) compared to non-disabled nurses (93.6%), with an overall weighted disability prevalence of 4.7%.

**Outcome Measures**: The primary measures of income used was the annual wages, which is the pre-tax wage and salary income (money received as an employee) for the previous year. Sources of income include wages, commissions, tips, cash bonuses and other income received from the employer. Income was inflation adjusted to 2023 US dollars, and the top and bottom 2.5% values were winsorized to remove outliers. Hourly wages calculated by dividing annual wages by total hours worked in the year. Total hours worked as calculated by multiplying hours worked per week and weeks worked in the year. The ACS does not ask respondents to report full-time versus part-time work.

**Statistical Software:** Analyses were conducted using Stata 18.0.

**Ethical Considerations:** This study was deemed exempt from IRB review.

**References (Supplementary Material):**

1. Ruggles S, Flood S, Sobek M, Brockman D, Cooper G, Richards S, Schouweiler M. IPUMS USA: Version 13.0 [dataset]. Minneapolis, MN: IPUMS, 2023.

<https://doi.org/10.18128/D010.V13.0>
2. Hall JP, Kurth NK, Ipsen C, Myers A, Goddard K. Comparing Measures Of Functional Difficulty With Self-Identified Disability: Implications For Health Policy. Health Aff (Millwood). 2022 Oct;41(10):1433-1441. doi: 10.1377/hlthaff.2022.00395. PMID: 36190890; PMCID: PMC10353341.

**Appendix 2:**

**Table S1. Characteristics of employed US nurses ages 20-65 by disability status, 2008-2023 (unadjusted and weighted^a^)**

| Variable | No Disability  (n= 456,425) | Any Disability^b^  (n= 17,425) | *P* value |
| --- | --- | --- | --- |
| Age, mean (SD), y | 42.6 (11.6) | 48.6 (11.4) | <.001 |
| Age Groups, No. (%) |  |  |  |
| 20-34 | 124,255 (29.7%) | 2333 (15.2%) | <.001 |
| 35-49 | 169,382 (38.5%) | 4865 (30.5%) |  |
| 50-65 | 162,788 (31.8%) | 10,227 (54.3%) |  |
| Sex, No. (%) |  |  |  |
| Male | 48,092 (11.1%) | 2138 (12.7%) | <.001 |
| Female | 408,333 (88.9%) | 15,287 (87.3%) |  |
| Race, No. (%) |  |  |  |
| American Indian or Alaska Native | 2064 (0.4%) | 142 (0.7%) | <.001 |
| Asian or Pacific Islander | 42,254 (9.4%) | 962 (5.5%) |  |
| Black/African American | 34,548 (10.5%) | 1557 (11.4%) |  |
| White | 357,704 (74.8%) | 13,763 (76.3%) |  |
| Multiracial | 13,836 (3.3%) | 772 (4.5%) |  |
| Other race, nec^d^ | 6019 (1.6%) | 229 (1.6%) |  |
| Ethnicity, No. (%) |  |  |  |
| Hispanic | 26,468 (6.8%) | 1079 (7.1%) | <.001 |
| Cuban | 2217 (0.6%) | 73 (0.5%) | <.001 |
| Mexican | 13,551 (3.5%) | 516 (3.4%) |  |
| Puerto Rican | 3367 (0.9%) | 195 (1.2%) |  |
| Other Hispanic^e^ | 7333 (1.9%) | 295 (2.0%) |  |
| Not Hispanic | 429,957 (93.2%) | 16,346 (93.0%) |  |
| Disability Type, No. (%)^f^ |  |  |  |
| Cognitive | NA | 2125 (13.0%) |  |
| Mobility | NA | 4215 (23.9%) |  |
| Independent living | NA | 596 (3.5%) |  |
| Self-care | NA | 294 (1.7%) |  |
| Vision | NA | 3046 (17.2%) |  |
| Hearing | NA | 3995 (22.1%) |  |
| More than 1 disability type | NA | 3154 (18.7%) |  |
| Occupation, No. (%)^g^ |  |  |  |
| Registered Nurses | 376,683 (83.6%) | 14,810 (85.5%) | <.001 |
| Nurse anesthetists | 4448 (0.9%) | 109 (0.7%) |  |
| Nurse practitioners and Nurse midwives | 24,088 (5.1%) | 710 (4.1%) |  |
| Top 3 industries |  |  |  |
| Hospitals | 290,247 (63.7%) | 9626 (55.1%) | <.001 |
| Outpatient care centers, physician offices | 52933 (11.4%) | 1913 (11.1%) |  |
| Nursing care facilities | 29169 (6.4%) | 1540 (8.7%) |  |
| Usual hours worked per week, mean (SD) | 38.06 (9.4) | 38.63 (10.4) | <.001 |
| Weeks worked last year, No. (%) |  |  |  |
| 1-13 weeks | 6860 (1.5%) | 446 (2.8%) | <.001 |
| 14-26 weeks | 7967 (1.8%) | 438 (2.6%) |  |
| 27-39 weeks | 12137 (2.6%) | 636 (3.7%) |  |
| 40-47 weeks | 17946 (3.8%) | 813 (4.6%) |  |
| 48-49 weeks | 7836 (1.6%) | 282 (1.6%) |  |
| 50-52 weeks | 403679 (88.7%) | 14810 (84.8%) |  |
| Annual Wages^h^, mean (SD), $^i^ | 80331.5 (38245.1) | 76510.2 (38437.7) | <.001 |
| Hourly Wages ^j^, mean (SD), $ | 43.9 (19.7) | 42.3 (20.1) | <.001 |

Abbreviations: NA, Not applicable

^a^ Weighting was done for means and standard deviations for continuous variables, and for percentages only for categorical variables. The counts for categorical variables are unweighted. All percentages for columns ‘No Disability’ and ‘Any Disability’ are column percentages.

^b^ Any Disability is defined as answering ‘Yes’ to any of the six disability questions in the American Community Survey related to: vision (blind or serious difficulty seeing even with glasses), hearing (deaf or serious difficulty hearing), cognitive (difficulty concentrating, remembering or making decisions), mobility (serious difficulty walking or climbing stairs), self-care (difficulty dressing or bathing), or independent living (difficulty doing errands).

^c^ Other Asians include Taiwanese, Filipino, Korean, Vietnamese, Bhutanese, Mongolian, Nepalese, Cambodian, Hmong, Laotian, Thai, Burmese, Indonesian, Malaysian and other Asians not included in above categories.

^d^ Other nec: all other races not elsewhere classified in the ACS

^e^ Other Hispanic includes Costa Rican, Guatemalan, Honduran, Nicaraguan, Panamanian, Salvadoran, Argentinian, Bolivian, Chilean, Colombian, Ecuadorian, Paraguayan, Peruvian, Uruguayan, Venezuelan, Spaniard, Dominican and other Hispanic ethnicities not included in above categories.

^f^ Disability types include respondents identifying as having only the one type of listed disability. Respondents with more than one disability type are included under ‘More than 1 disability’ type.

^g^ ACS started dividing nursing professionals into ‘Registered Nurses’, ‘Nurse anesthetists’, and ‘Nurse practitioners and Nurse midwives’ starting from 2010. These data reflect years 2010 to 2023.

^h^ Annual wage denotes income earned from wages as an employee for the previous year. It is the total pre-tax wage and salary income. Sources of income include wages, salaries, commissions, cash bonuses, tips, and other money income received from an employer.

^i^ All $ values adjusted to 2023 US dollars

^j^ Hourly wages calculated by dividing annual wages by total hours worked in the year. Total hours worked is calculated by multiplying hours worked per week and weeks worked in the year.

**Table S2. Differences in earnings and total hours worked for employed nurses with a disability compared to nurses without a disability, additionally adjusted for industry^a^**

|  | Adjusted Models | | | Models Additionally Adjusting for Total Hours Worked | | |
| --- | --- | --- | --- | --- | --- | --- |
|  | Estimate | 95% CI | P value | Estimate | 95% CI | *P* value |
| Difference in Annual Wages ^b^, percentage points ^c^ | -11.1 | -12.5, -9.8 | <.001 | -9.25 | -10.4, -8.14 | <.001 |
| Difference in Total hours worked (annual) ^d^ | -30.0 | -42.1, -18.0 | <.001 | NA | NA | NA |
| Difference in Hourly Wages ^e^, percentage points ^c^ | -6.4 | -7.29, -5.51 | <.001 | -6.7 | -7.6, -5.8 | <.001 |

^a^ All outcomes were analyzed for employed nurses aged 20-65, using linear regression models with robust standard errors. Models were adjusted for fixed effects for age (individual years), sex (male, female), race (White, Black/African American, American Indian or Alaskan Native, Asian or Pacific Islander, Multiracial, Other), ethnicity (Hispanic, Not Hispanic), state and metropolitan area, and survey year.

^b^ Annual wage denotes income earned from wages as an employee for the previous year. It is the total pre-tax wage and salary income. Sources of income include wages, salaries, commissions, cash bonuses, tips, and other money income received from an employer.

^c^ Because logged wages was used as the dependent variable, estimated earnings disparities can be expressed as percent differences (coefficient multiplied by 100).

^d^ Total hours in a year worked is calculated by multiplying hours worked per week and weeks worked in the year.

^e^ Hourly wages calculated by dividing annual wages by total hours worked in the year. Total hours worked is calculated by multiplying hours worked per week and weeks worked in the year.

**Appendix 3: Additional References in Manuscript**

6. Moore J, Continelli T. Racial/Ethnic Pay Disparities among Registered Nurses (RNs) in U.S. Hospitals: An Econometric Regression Decomposition. *Health Serv Res*. 2016;51(2):511-529. doi:10.1111/1475-6773.12337

7. Jackson BL, Cameron VK, Hodgens TM, et al. Disability and accommodation use in US bachelor of science in nursing programs. *JAMA Netw Open*. 2025;8(2):e2461038. doi:[10.1001/jamanetworkopen.2024.61038](https://doi.org/10.1001/jamanetworkopen.2024.61038)

8. Sheets ZC, Nouri Z, Conrad SS, et al. Workplace Accommodations and Attrition Among Physicians With Disabilities. JAMA Netw Open. 2026;9(3):e261922. doi:10.1001/jamanetworkopen.2026.1922

9. Kutney-Lee A, Stimpfel AW, Sloane DM, Cimiotti JP, Quinn LW, Aiken LH. Changes in patient and nurse outcomes associated with magnet hospital recognition. *Med Care*. 2015;53(6):550-557. doi:10.1097/MLR.0000000000000355

10. Yu H, Bonett S, Flores DD, Meanley S, Choi SK, Hanneman T, Bauermeister JA. The relationship between a hospital's Magnet status and LGBTQ+ inclusivity in policies and practices in US hospitals. *Res Nurs Health*. 2025;48:30-40. doi:10.1002/nur.22422

11. Muir KJ, Maye A, McHugh MD, Aiken LH, Vo V, Lasater KB. Virtual Nursing for the Care of Hospitalized Patients. *JAMA Netw Open*. 2025;8(12):e2545597. Published 2025 Dec 1. doi:10.1001/jamanetworkopen.2025.45597
